# Immunomodulatory Effects of the Antimicrobial Peptide KR-20: Implications for Trichomoniasis

**DOI:** 10.3390/molecules31030413

**Published:** 2026-01-26

**Authors:** María G. Ramírez-Ledesma, Eva E. Ávila, Nayeli Alva-Murillo

**Affiliations:** 1 Departamento de Neurobiología Celular y Molecular, Universidad Nacional Autónoma de México, Boulevard Juriquilla #3001, Querétaro PC 76230, Mexico; lu.rmzledesma@gmail.com; 2 Departamento de Biología, DCNE, Universidad de Guanajuato, Colonia Noria Alta, Guanajuato City PC 36050, Mexico

**Keywords:** trichomoniasis, KR-20, monocytes

## Abstract

Trichomoniasis is the most prevalent non-viral sexually transmitted infection worldwide and is caused by *Trichomonas vaginalis*. The development of resistance against the standard treatment, metronidazole, highlights the need for alternative therapeutic approaches. The role of innate immune cells is crucial for understanding trichomoniasis; however, the contribution of monocytes remains poorly characterized. We previously reported that the antimicrobial peptides LL-37 and its derivative KR-20 are trichomonacidal. In other systems, LL-37 displays immunomodulatory effects. Nevertheless, whether these peptides modulate monocyte responses in the presence of *T. vaginalis* remains unknown, which was the aim of this study. U937 monocytes were co-incubated with LL-37 or KR-20 (3 h), with or without parasite. Monocyte metabolic activity, nitric oxide production, and relative expression of innate immune genes were assessed. LL-37 decreased monocyte metabolic activity and upregulated TNF-α expression (10 and 5 μM, respectively) in parasite-challenged monocytes. Meanwhile, KR-20 (2.5–10 μM) preserved metabolic activity, bound microbial components (LPS), reduced parasite-induced nitric oxide production, and downregulated the expression of IL-8, TNF-α, IL-1β, and COX-2 in infected monocytes. This work provides initial evidence that KR-20 modulates innate immune response in monocytes during *T. vaginalis* infection, suggesting its potential—yet to be fully validated—as an immunomodulatory candidate for trichomoniasis.

## 1. Introduction

Sexually transmitted infections (STIs) have a profound impact on reproductive and sexual health. Among these, trichomoniasis is the most prevalent nonviral STI worldwide, even more common than gonorrhea or chlamydia. *Trichomonas vaginalis*, an anaerobic flagellated protozoan parasite, is the etiological agent of trichomoniasis, with humans being the only known host [1]. The World Health Organization (WHO) estimates the prevalence of trichomoniasis to be 5.3% among women and 0.6% among men [2]. The asymptomatic manifestation of this STI is very frequent [3], which could impact on the actual prevalence, diagnosis, parasite persistence in the genital tract, and transmission. It has been suggested that trichomoniasis is associated with serious health consequences such as adverse pregnancy outcomes [4] and pelvic inflammatory disease [5,6] in women. Whilst in men, this STI is linked to infertility [7] and prostate cancer, though the latter is not conclusive [8]. For both, there is a higher risk of acquiring human immunodeficiency virus (HIV) and other STIs [9].

*T. vaginalis* spreads mainly through sexual intercourse and infects the urogenital tract, where it can persist for extended periods [10]. First, the parasite—via mucinases— breaks down a mucous layer and crosses it [11]. Then, *T. vaginalis* attaches to genital epithelial cells through the parasite’s surface lipoglycan, which binds to host galectin-1 [12]. This adherence is also mediated by parasite membrane proteins (i.e., TvBAP1) [13], thus preventing its elimination through gravity and secretions [3]. Furthermore, *T. vaginalis* secretes exosomes that contribute to adherence [10], and some strains are cytotoxic, extracting nutrients from the damaged cells [3,14]. *T. vaginalis* can harbor symbionts such as *Trichomonas vaginalis* virus (TVV) and *Mycoplasma hominis*, which might contribute to pathogenesis and influence host immune response [3].

The female genital innate immune defense against *T. vaginalis* has been studied more extensively than the male defense against this parasite. It begins when epithelial cells secrete IL-6, IL-8, macrophage inflammatory protein (MIP)-3α, monocyte chemoattractant protein-1 (MCP-1), and cyclooxygenase 2 (COX-2), among others, in response to parasite detection [15,16,17,18,19,20]. These molecules are associated with immune cell migration and activation, as well as in the inflammatory process [16,21,22,23,24]. Neutrophils dominate the immune response at the infection’s site, potentially influencing both parasite clearance and inflammation [25]. However, little is known about the role of other immune cells, such as monocytes. These cells might be essential for the early immune response to protozoan parasites because monocyte populations are in peripheral circulation and are recruited to sites of inflammation, where they could differentiate into inflammatory cells, such as monocyte-derived macrophages, and release nitric oxide (NO) and cytokines [26]. It has been reported that *T. vaginalis* induces IL-8 secretion by monocytes [27,28], thereby recruiting neutrophils. Furthermore, *T. vaginalis* stimulates the release of extracellular traps (ETs) in monocytes [29].

Metronidazole has been the classical treatment for trichomoniasis since 1959 [30]. Nowadays, tinidazole and secnidazole are also used [31]. However, clinical cases are resistant (~10%) [1,32], and reports indicate cross-resistance between 5-nitroimidazole drugs [33]. To address this challenge, our research group has focused on antimicrobial peptides (AMPs) [34]. These peptides are synthesized by prokaryotes and eukaryotes and possess microbicidal and immunomodulatory properties [35].

In this context, the human cathelicidin LL-37 has been studied due to its wide range of reported biological activities, such as its role as a chemoattractant for immune cells (monocytes and neutrophils), its regulation of cytokine levels, and its ability to neutralize toxins (LPS), among others [36]. Short fragments derived from LL-37 have also been studied. Notably, the KR-20 peptide (residues 18–37 of LL-37), which has been detected in human sweat, possesses microbicidal activity against *Candida albicans* (minimum inhibitory concentration, MIC = 10 µM), *Staphylococcus aureus* (MIC = 16 to >60 µM), *Escherichia coli* (MIC = 60 µM), *Acinetobacter baumannii* (MIC = 6.5–30 µM), and *Entamoeba histolytica* (10 and 50 µM) [37,38,39]. Previously, we reported that LL-37 and its derivatives (KR-20, FK-13-NH_2_, and KR-12) affected the viability of both a metronidazole-sensitive and a metronidazole-resistant *T. vaginalis* strain as early as 3 h [34]. Among them, KR-20 was the most effective, with MIC_50_ values of 4.8 µM and 7.8 µM, respectively. To our knowledge, the immunomodulatory effect of LL-37 and KR-20 on monocytes in trichomoniasis has not been evaluated, which was the aim of this study. We determined that human cathelicidin LL-37 decreased the monocyte metabolic activity in a concentration-dependent manner, reversed the parasite-induced NO production and *IL-8* gene expression, but increased the *TNF-α* gene expression in *T. vaginalis*-challenged monocytes. Moreover, we observed that KR-20 modulates the immune response of U937 monocytes during *T. vaginalis* infection by reducing parasite-induced NO production and downregulating proinflammatory gene expression, without affecting monocyte metabolic activity. Therefore, KR-20 is suggested to have therapeutic potential against trichomoniasis.

## 2. Results

### 2.1. The Antimicrobial Peptide KR-20 Does Not Affect the Metabolic Activity of the U937 Monocyte

The effect of LL-37 and KR-20 peptides on monocyte metabolic activity, indicative of viability [40], has not been previously determined. To address this, we used a peptide concentration range of 2.5–10 µM. Monocytes were incubated for 3 h in the presence or absence of *T. vaginalis* (GT-13) [41]. The 3 h incubation period was selected based on our previous findings showing that these peptides exert trichomonacidal effect after 3 h of exposure [34], and on reports indicating that *T. vaginalis* triggers an early innate immune response in immune cells within 2–6 h of interaction [42,43].

**Figure 1:**
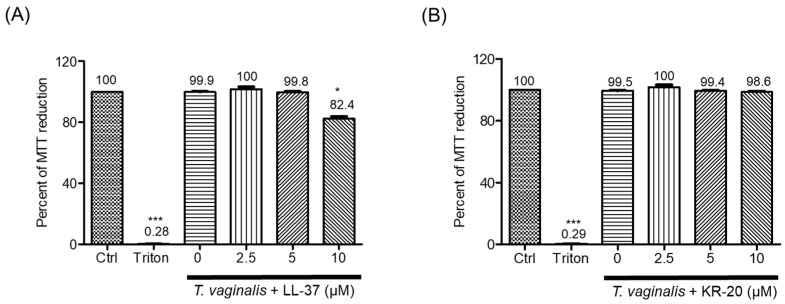
Metabolic effect of antimicrobial peptides LL-37 and KR-20 on monocyte activity. U937 monocytes were cultured with (**A**) LL-37 or (**B**) KR-20, as well as *T. vaginalis*. After a 3 h interaction, metabolic activity was determined using the MTT assay. Kruskal–Wallis statistical analysis was carried out, comparing each experimental condition against basal (unstimulated) cells. Four independent experiments were performed in triplicate. Data represents mean values. * *p* < 0.05, *** *p* < 0.001.

The challenge with *T. vaginalis* (multiplicity of infection, MOI, of 10 monocytes: 1 trophozoite) did not significantly alter monocyte metabolic activity compared with untreated controls (Ctrl) (Figure 1). However, exposure to 10 µM LL-37 for 3 h reduced U937 monocyte metabolic activity by 16.4% (Figure S1A). Interestingly, this effect was also evident in *T. vaginalis*-challenged monocytes, where 10 µM LL-37 decreased the metabolic activity by 17.6% (Figure 1A). In contrast, neither KR-20 alone nor the tripartite interaction of KR-20, *T. vaginalis*, and monocytes (MOI of 10:1) affected monocyte metabolic activity at any of the tested concentrations (2.5, 5 and 10 µM) (Figure S1B and Figure 1B). As expected, the negative control (Triton X-100, 1%) nearly abolished monocyte metabolic activity (~0.2%). For this reason, the concentration of 10 µM LL-37 was not included in further assays. Overall, these results indicate that KR-20 peptide does not affect monocyte metabolic activity in our experimental conditions, whereas LL-37 reduces it at higher concentrations (10 µM).

### 2.2. T. vaginalis Does Not Induce the Release of Extracellular Traps by U937 Monocytes

**Figure 2:**
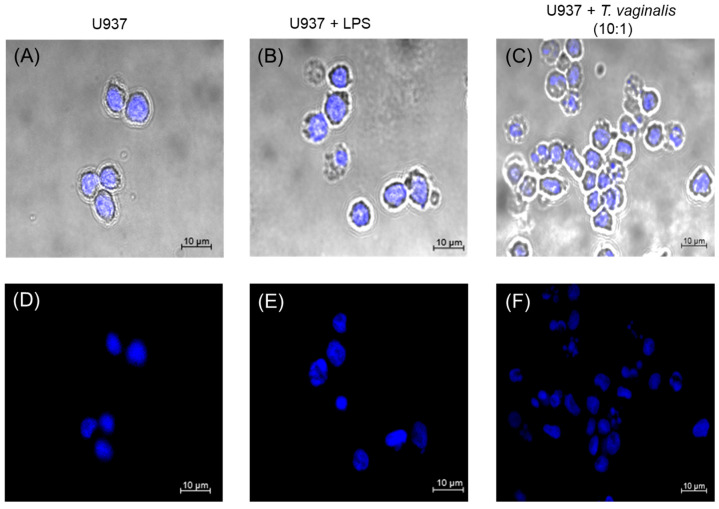
Stimulation of U937 cells with LPS and *T. vaginalis*. (**A**–**C**) Phase-contrast splicing with fluorescence; (**D**–**F**) fluorescence; (**A**,**D**) unstimulated U937 cells; (**B**,**E**) U937 cells stimulated with LPS (1 μg/mL, 3 h); (**C**,**F**) monocytes stimulated with *T. vaginalis* (MOI of 10:1, 3 h). Blue, Hoechst 33342. 40× objective with immersion oil.

We investigated whether the *T. vaginalis* strain GT-13 induces the release of extracellular traps (ETs) by U937 monocytes. For this, monocytes were exposed to trophozoites at a MOI of 10:1 for 3 h, stained with Hoechst 33342, and analyzed by confocal microscopy. In unstimulated U937 monocytes, micrographs revealed the typical morphology previously described [44], with no evidence of extracellular DNA fibers stained (Figure 2A,D). Similarly, neither *T. vaginalis*-challenged nor LPS-stimulated monocytes exhibited ET formation, as extracellular DNA fibers were absent in all conditions (Figure 2B,E,C,F). These findings suggest that monocyte integrity remains preserved during the interaction with *T. vaginalis*, as there was no release of extracellular traps—an event that could potentially trigger inflammatory responses.

### 2.3. The KR-20 Peptide Regulates Nitric Oxide Production

**Figure 3:**
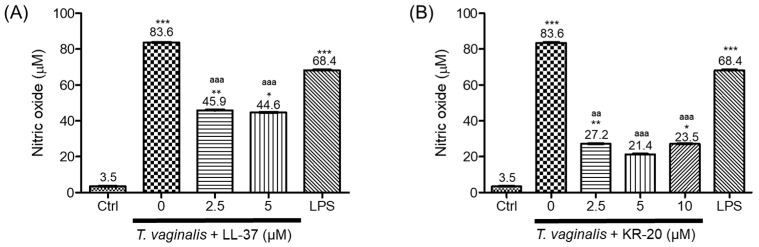
Nitric oxide production by monocytes co-incubated with antimicrobial peptides and *T. vaginalis*. U937 cells were incubated for 3 h at 37 °C with *T. vaginalis* (MOI of 10:1) in the presence of (**A**) LL-37 or (**B**) KR-20. The supernatants were collected, and nitrite levels were quantified using the Griess method. Each bar represents the mean ± SE of four independent experiments performed in triplicate. Statistical analysis was performed using the Kruskal–Wallis, comparing each condition with either unstimulated (basal) cells or with *T. vaginalis*-stimulated controls. Significant differences relative to basal cells (Ctrl) are indicated as follows: * *p* < 0.05, ** *p* < 0.01, *** *p* < 0.001. Significant differences compared with *T. vaginalis*-stimulated controls are indicated as follows: ^aa^ *p* < 0.01, ^aaa^ *p* < 0.001.

We next assessed whether the LL-37 or its derivative KR-20 modulates nitric oxide (NO) production in monocytes, either in the presence or absence of *T. vaginalis*. For this, the quantification of nitrites was measured by the Griess reaction. Exposure to *T. vaginalis* (MOI of 10:1, 3 h) markedly elevated nitrite production (83.6 µM) compared to basal levels (3.5 µM) (Figure 3). LL-37 alone produced a modest concentration-dependent increase in nitrite production (17.2–20.1 µM; Figure S2A) but attenuated the parasite-induced elevation when co-incubated with *T. vaginalis* (Figure 3A). Similarly, KR-20 alone upregulated nitrite production by U937 monocytes (10.3–38.4 µM; Figure S2B). Nonetheless, co-incubation of KR-20 with trophozoites reversed this effect, resulting in nitrite concentrations of 27.2, 21.4, and 23.5 µM at peptide concentrations of 2.5, 5, and 10 µM, respectively (Figure 3B). Interestingly, this inhibitory effect was more pronounced for KR-20 than for LL-37. As expected, LPS (1 µg/mL, 3 h) augmented nitrite concentration (68.4 µM, Figure 3). Collectively, these results indicate that both peptides modulate NO production in U937 monocytes.

### 2.4. Neutralization of Microbial Components by the KR-20 Antimicrobial Peptide

**Figure 4:**
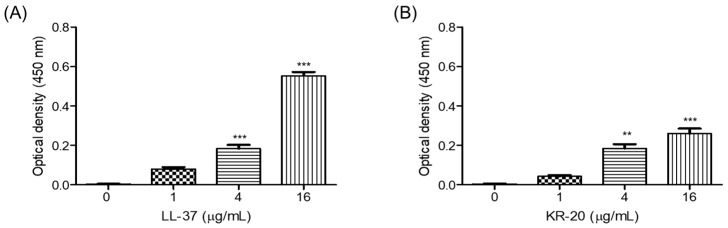
Binding of the antimicrobial peptides to bacterial LPS. The binding capacity of antimicrobial peptides—(**A**) LL-37 or (**B**) KR-20—to LPS was assessed by ELISA. Antimicrobial peptide concentrations ranging from 0 to 16 μg/mL were employed. The data corresponds to four duplicate experiments. Statistical analysis was conducted using the Kruskal–Wallis method, comparing each peptide concentration with its corresponding baseline (0 μg/mL). Significant differences are indicated as follows: ** *p* < 0.01, *** *p* < 0.001.

Using an indirect ELISA, we found that the KR-20 peptide binds to the microbial component LPS in a concentration-dependent manner, with detectable binging starting at 4 µg/mL (1.62 µM) and reaching its maximum at 16 µg/mL (6.48 µM) (Figure 4B). In agreement with previous reports [45], LL-37 also bound to LPS within the same concentration range (4–16 µg/mL or 0.89–3.56 µM), displaying a stronger interaction than KR-20 (Figure 4). These results suggest that the KR-20 retains the ability to interact with LPS, albeit to a lesser extent than LL-37, suggesting its potential to attenuate proinflammatory responses triggered by microbial components.

### 2.5. KR-20 Downregulates the Relative Expression of Innate Immune Genes in T. vaginalis-Challenged Monocytes

**Figure 5:**
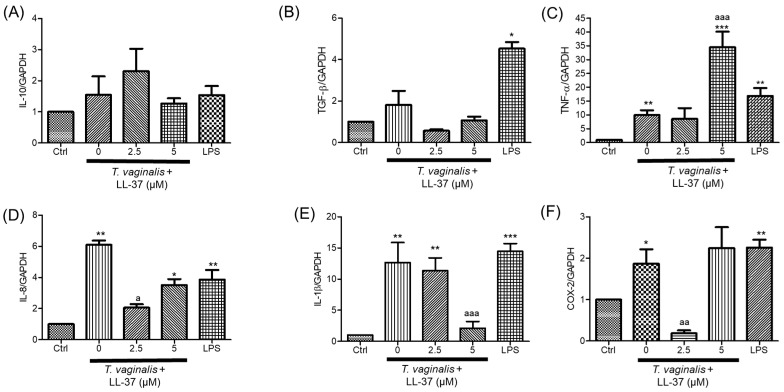
Effect of LL-37 on innate immune gene expression in U937 monocytes stimulated with *T. vaginalis*. U937 cells were incubated for 3 h at 37 °C with LL-37 (2.5 or 5 μM) and *T. vaginalis* (MOI of 10:1). LPS (1 μg/mL) was used as a positive control. Gene expression levels were quantified using the ΔΔCt method and normalized to *GAPDH*. Panels represent (**A**) *IL-10*, (**B**) *TGF-*β, (**C**) *TNF-α*, (**D**) *IL-8*, (**E**) *IL-1β*, and (**F**) *COX-2*. Data corresponds to the mean ±SE of three experiments, each performed in triplicate. The results were analyzed using the Kruskal—Wallis test. The symbol “*” indicates significant differences in relation to unstimulated cells (* *p* < 0.5, ** *p* < 0.01, *** *p* < 0.001). The symbol “^a^” indicates significant differences compared with *T. vaginalis*-stimulated control cells (^a^ *p* < 0.05, ^aa^ *p* < 0.01 ^aaa^ *p* < 0.001).

**Figure 6:**
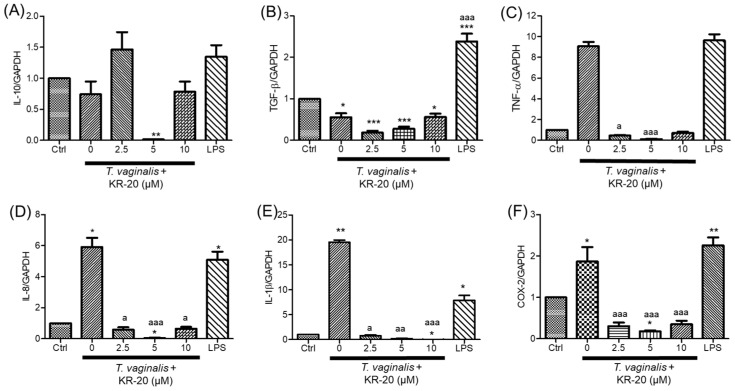
Effect of KR-20 on innate immune gene expression in monocytes stimulated with *T. vaginalis*. U937 cells were incubated for 3 h at 37 °C with KR-20 (2.5, 5, or 10 μM) in the presence or absence of *T. vaginalis* (MOI of 10:1). LPS (1 μg/mL) was included as a positive control. Gene expression levels were quantified using the ΔΔCt method and normalized to *GAPDH*. Panels correspond to (**A**) *IL-10*, (**B**) *TGF-β*, (**C**) *TNF-α*, (**D**) *IL-8*, (**E**) *IL-1β*, and (**F**) *COX-2*. Values represent the mean ± SE from three independent experiments conducted in triplicate. Statistical analysis was performed using Kruskal–Wallis test. The symbol “*” indicates significant differences in relation to unstimulated cells (* *p* < 0.5, ** *p* < 0.01, *** *p* < 0.001). The symbol “^a^” indicates significant differences compared with cells stimulated with *T. vaginalis* (^a^ *p* < 0.05, ^aa^ *p* < 0.01, ^aaa^ *p* < 0.001).

We evaluated the effects of LL-37 or KR-20 peptides on the relative expression of innate immune genes in U937 monocytes, either infected or uninfected with *T. vaginalis*. In parasite-challenged monocytes *IL-8*, *TNF-α*, *IL-1β*, and *COX-2* mRNA levels were increased (~6-, ~9, and ~20-, ~2-fold, respectively); however, *IL-10* and *TGF-β* mRNA levels remained statistically comparable to control values (Figure 5 and Figure 6). Treatment with LL-37 upregulated *IL-10*, *TNF-α*, *IL-8*, and *COX-2* gene expression, reaching ~13-, ~16-, ~4-, and ~5-fold increases at 5 µM, respectively (Figure S3A,C,D,F). Conversely, LL-37 attenuated the *T. vaginalis*-induced upregulation of *IL-8*, and *IL-1*β, but further enhanced *TNF-α* (34.7-fold, respectively) in infected monocytes (Figure 5C–E). Regarding *COX-2*, only 2.5 µM LL-37 reduced its mRNA levels in *T. vaginalis*-challenged monocytes (Figure 5F).

In contrast, KR-20 treatment (2.5, 5, and 10 µM, 3 h) generally downregulated or did not modify the gene expression of *IL-8*, *TNF-α*, *IL-1β*, *IL-10*, *TGF-*β, and *COX-2* compared with untreated controls (Figure S4). Notably, 2.5 µM KR-20 increased *TGF*-ꞵ mRNA levels (10.7-fold, Figure S4B). Importantly, KR-20 peptide markedly reduced the *T. vaginalis*-induced expression of *TNF-α*, *IL-8*, *IL-1β*, and *COX-2* (Figure 6C–F). As expected, *IL-8*, *TNF-α*, *IL-1*β, *TGF*-β and *COX-2* mRNA levels were induced in LPS-treated monocytes compared to basal levels (Figure 5 and Figure 6). Our data show that KR-20 (2.5–10 µM) consistently attenuated the transcription of proinflammatory mediators evaluated. Although LL-37 (2.5 µM) also reduced some inflammatory markers, the overall effect was less pronounced. These findings suggest that KR-20 exerts anti-inflammatory properties in this model, supporting its relevance as a promising modulator of monocyte responses during *T. vaginalis* challenge.

To assess whether the innate immune gene expression induced by *T. vaginalis* in monocytes could be influenced by its symbionts, we evaluated the presence of *M. hominis* and TVV (TVV1-TVV4) in the GT-13 strain. Neither *M. hominis* nor any TVV species were detected (Figures S5 and S6). These results confirm that the immune responses observed in U937 monocytes arise solely from the interaction with *T. vaginalis* itself, strengthening the interpretation of the KR-20-mediated effects by that the parasite-associated symbionts do not influence them.

## 3. Discussion

The limited treatment options for trichomoniasis increase the risk of chronic infections and transmission, posing a potential public health issue. Searching new alternative treatments, we previously demonstrated that human cathelicidin LL-37 and its shorter derivative peptide KR-20 (2–50 µM, 24 h) decreased the growth of *T. vaginalis* (GT-13 strain), with a minimum inhibitory concentration 50 (MIC_50_) calculated at 12.9 and 4.8 µM, respectively, with KR-20 showing greater effectiveness [34]. Antimicrobial peptides (AMPs) are molecules of great interest due to their microbicidal and immunomodulatory activity [46]. The antimicrobial peptide LL-37 is known to possess these activities [36]. While KR-20 has been reported to exhibit antimicrobial activity, the potential for KR-20 or its parent peptide to modulate the monocyte immune response in the context of trichomoniasis has not been investigated. Therefore, this study aimed to analyze the immunomodulatory effects of both peptides on monocytes infected with or not infected by *T. vaginalis*.

LL-37 exhibits pleiotropic effects due to its ability to act through multiple mechanisms that depend mainly on the surrounding microenvironment and peptide concentration [47,48]. Indeed, LL-37 has been reported to alter metabolic activity or even to display cytotoxicity toward certain mammalian cells [49], such as human osteoblast-like MG63 cells (1–9 µM for 3–4 h) [50,51], monocytes (9 µM, 4 h) [51], keratinocytes (22 µM, 24 h) [52], and lung epithelial cells (11 and 22 µM, 6 h) [53], among others. In contrast, at lower concentrations, LL-37 does not compromise the viability of human blood-derived monocytes (2.2 to 11 µM, up to 4 h) [54], THP-1 monocytes (0.3–3 µM, 4 h) [51] or lung epithelial cells (2.2–6.6 µM, 6 h) [53], and can even protect peripheral mononuclear cells from apoptosis (2.2 µM, 72 h) [55]. In agreement with this dual behavior, our results showed that LL-37 decreased the metabolic activity—suggestive of cell viability—of U937 monocytes only at the highest concentration tested (10 µM, 3 h), while lower concentrations (2.5 and 5 µM) had no effect (Figure 1A and Figure S1A). LL-37 may cause cell death or reduces cell viability through different pathways, such as caspase-independent [50,56] or -dependent apoptosis [57], as well as necrosis [58], which depends on peptide concentration, cell type, and exposure time; however, further experiments are needed to clarify the mechanism in our model.

In contrast to LL-37, the derivative peptide KR-20 did not affect monocyte metabolic activity at any of the concentrations evaluated, either in unstimulated cells or in parasite-challenged monocytes (Figure 1B and Figure S1B). This observation is consistent with previous reports showing that KR-20 does not affect the integrity of human erythrocytes (≤100 µM) [34,39] nor does it reduce the metabolic activity of human fibroblast (50 µM, 24 h) [34]. These results suggest that KR-20 exhibits a more favorable safety profile than LL-37. Further, this is supported by studies demonstrating that removal of N-terminal residues from LL-37 generates derivatives with reduced hemolytic and cytotoxic effects while maintaining structural stability and antimicrobial function [52,59]. KR-20, composed of residues 18–37 of LL-37, aligns with this strategy. Our data reinforces this concept and highlights the potential of KR-20 as a safer, alternative, anti-parasitic peptide without compromising host metabolic activity.

Monocytes act as first-line defense cells, like other immune cells, and can deploy multiple early defense mechanisms, including the formation of extracellular traps (ETs) [60], which contribute to pathogen control but can also amplify inflammatory responses [61]. ET release has been reported in *T. vaginalis*-stimulated TPH1 monocytes, suggesting that this protozoan can activate DNA-based defensive structures [29]. Given that the central aim of our study was to determine whether the KR-20 peptide modulates the monocyte response during *T. vaginalis* infection, it was essential first to establish whether U937 cells form ETs under our experimental conditions. In contrast to what was reported [29], extracellular traps were not detected when U937 monocytes interacted with trophozoites of the GT-13 strain (Figure 2C,F). This discrepancy may be attributed to differences in the monocyte cell line, *T. vaginalis* strain, MOI, or interaction time. The absence of ETs formation in our model is relevant for interpreting the immunomodulatory effects of KR-20. Since ETs release can strongly influence inflammatory responses [62], their absence indicates that the changes observed in cytokine gene expression are not a consequence of ETs-mediated cell activation but instead reflect a direct modulation of monocytes responses by *T. vaginalis* and the peptides.

*T. vaginalis* can harbor endosymbionts -including four types of *Trichomonas* virus (TVV) as well as *Mycoplasma hominis* and *Mycoplasma girerdii*—all of which are known to influence parasite virulence and modulate host inflammatory responses [63,64,65]. Clinical studies have shown that infections involving TVV+ strains are associated with more severe symptoms [66], and TVV-bearing parasites elicit stronger proinflammatory cytokine responses in host cells [67]. In monocytes, *T. vaginalis* harboring *M. hominis* induces higher expression of proinflammatory cytokines compared to a mycoplasma-free strain [68,69,70]. For these reasons, confirming the absence of viral and mycoplasma symbionts in GT-13 strain was essential to accurately attribute the immunological effects observed in this study. Our analyses confirmed that neither viruses nor *Mycoplasma* sp. were found in the parasite used in this study (Figures S5 and S6). These results indicate that the change in monocyte immune response reported here reflects the direct interaction between monocytes, *T. vaginalis*, and peptides, without confounding contributions from endosymbionts.

Monocytes are involved in the production of nitric oxide (NO), a proinflammatory mediator that plays a crucial role in the host immune response against pathogens, such as parasites [71]. Previous reports have shown that *T. vaginalis* can induce NO synthesis in human monocyte-derived macrophages, contributing to the inflammation characteristic of trichomoniasis [72]. Furthermore, under iron-deficient conditions, the parasite can survive by increasing NO [73]; therefore, it is crucial to regulate the high concentrations of this molecule induced by the parasite in the microenvironment. In our model, U937 monocytes stimulated for 3 h with *T. vaginalis* (MOI of 10:1) increased NO levels—24-fold above unstimulated cells- and even exceeding the response elicited by LPS, a canonical inducer (Figure 3) [74]. NO production is largely mediated by inducible nitric oxide synthase (iNOS), whose transcription is regulated by transcription factors, such as NF-κB, STAT-1, and IRF-1 [75]. In particular, NF-κB could be activated upon Toll-like receptor (TLR) engagement [75]. In the context of trichomoniasis, the inflammation response has been associated with several TLRs, including TLR2, TLR3, TLR4, and TLR5, leading to downstream activation of NF-κB in host immune cells [76,77]. This aligns with our observation that *T. vaginalis* is an inducer of NO production in U937 cells, and this is likely due to the above mechanistic framework.

Human cathelicidin LL-37 is known for its immunomodulatory properties, including the ability to exert anti-inflammatory effects in various cellular models. Several studies have shown that LL-37 can attenuate NO production by reducing *iNOS* expression triggered by inflammatory stimuli [78,79,80,81]. Consistent with these reports, LL-37 decreased NO levels when added to U937 monocytes stimulated with the parasite (Figure 3A). LL-37 (4.5 µM, 1 h) has also been shown to inhibit NF-κB nuclear translocation and suppress TLR-mediated activation in LPS-treated human monocytes [82], which may account -in part- for the reduced NO production in our model. Interestingly, its derivative peptide KR-20 not only preserved this anti-inflammatory activity of LL-37 but also exerted a more pronounced reduction in NO production in *T. vaginalis*-stimulated monocytes (Figure 3B [75]). To our knowledge, this is the first report demonstrating the ability of the KR-20 peptide to modulate an innate immune component—specifically, nitric oxide production—in monocytes. These findings highlight KR-20 as a promising immunomodulatory peptide with improved anti-inflammatory potency relative to LL-37, thereby underscoring the novelty and biological relevance of KR-20 within the context of the host response to *T. vaginalis*.

LL-37 possesses anti-endotoxin activity, as it can bind and neutralize microbial components (e.g., LPS), thereby dampening the host’s proinflammatory response, such as IL-8 and NO production [45,79,80]. However, whether KR-20 retains this property had not been previously determined. In this study, we demonstrated that KR-20 is also capable of binding LPS, although with lower affinity than LL-37 (Figure 4), demonstrating for the first time that this derivative peptide preserves the endotoxin-neutralizing capacity of its parent AMP. This may help explain, at least in part, the reduced NO production observed in *T. vaginalis*-stimulated monocytes treated with either LL-37 or KR-20 (Figure 3). Our findings indicate that KR-20 not only displays a distinct and safer immunomodulatory profile compared to LL-37 but also maintains the anti-endotoxin activity.

Trichomoniasis is typically accompanied by leukocyte infiltration and elevated levels of proinflammatory cytokines, and *T. vaginalis* is known to exploit this exacerbated inflammatory environment to promote tissue damage and establish infection [65]. Consistent with this pathogenic profile, several innate immune cells can sense *T. vaginalis*, thereby activating an inflammatory response [77]. In this sense, IL-8 secretion increases in *T. vaginalis*-challenged neutrophils, monocytes, or human vaginal cells [28,43,70,77,83]. *T. vaginalis* also induces the IL-1β secretion in monocytes and macrophages [69,70,72], while components of the parasite upregulate *IL-10* and *TNF-α* expression in macrophages [84]. Additionally, increased *TGF-β* expression has been reported in vaginal and cervical tissues in a murine model of infection [85]. Given this evidence, we focused on analyzing the relative expression of selected cytokines and chemokines (*IL-1β*, *TNF-α*, *IL-10*, *TGF-β*, and *IL-8*), as these represent some of the most consistently studied early innate immune mediators in both *in vitro* and *in vivo* models of *T. vaginalis* infection [27,28,72,77,85,86,87,88,89,90]. This study focused on evaluating the relative mRNA expression of innate immunity genes as an initial approach to characterize the monocyte response to antimicrobial peptides and *T. vaginalis*. Future studies will include protein quantification to confirm the functional consequences of these transcriptional changes.

We found that *T. vaginalis* significantly increased *IL-8*, *IL-1β*, and *TNF-α* gene expression in U937 monocytes, whereas *IL-10* and *TGF-β* transcript levels remained unchanged (Figure 5 and Figure 6). This aligns with previous reports, which describe that this parasite induces an inflammatory response. In this sense, some mechanistic pathways triggered by the parasite have been reported. For example, extracellular vesicles from *T. vaginalis* can enhance IL-8 and IL-1β production through TLR3 upregulation and NF-κB activation [77]. IL-8 may also be induced via TLR4 signaling, reactive oxygen species (ROS), MAPK pathways, and NF-κB activation [91]. Likewise, parasite-induced TNF-α production has been associated with PI3K/AKT and MAPK signaling cascades [92], while IL-1β secretion is promoted through ROS generation and NLRP3 inflammasome activation [93]. Although the molecular mechanisms underlying the inflammatory responses were not evaluated in this study, they represent an important perspective for future work.

Even though the role of COX-2 in trichomoniasis has been minimally explored—mainly in epithelial cells [18,19,20]—this enzyme is a key mediator of prostaglandin synthesis and a well-recognized regulator of inflammatory processes [24]. COX-2-dependent pathways have been implicated in the host response to several protozoan infections, and its inhibition has emerged as a potential therapeutic approach in parasitic disease. This work provides, to our knowledge, the first evidence that *T. vaginalis* stimulates *COX-2* expression in monocytes (Figure 5 and Figure 6). Given the limited understanding of COX-2 regulation during *T. vaginalis* infection and its potential relevance as a modulatory or therapeutic target, assessing its gene expression in monocytes provides valuable insight into the proinflammatory profile induced by the parasite and supports a deeper evaluation of host-directed intervention strategies. In this sense—in a non-inflammatory context—few studies have examined the effect of LL-37 on COX-2, reporting that activation of the P2X7R and FPR2 receptors by LL-37 induces *COX-2* expression [94,95], which is consistent with the findings of the present study (Figure S3F). Conversely, we observed that LL-37 (2.5 µM) and its derivative KR20 (2.5–10 µM) attenuated the parasite-induced upregulation of *COX-2* expression (Figure 5F and Figure 6F), indicating that the activity of antimicrobial peptides can shift depending on the inflammatory microenvironment.

Evidence has shown that LL-37 exerts an influence on the expression of anti- and proinflammatory molecules in various immune cell types, as well as a few LL-37 derivative peptides and their analogs [96,97,98,99], to maintain a fine balance in inflammatory response. In our model, we found that LL-37 upregulated the expression of *IL-10*, *TNF-α* and *IL-8* genes in U937 monocytes in a concentration-dependent manner (Figure S3). This finding aligns with a previous report, which showed that LL-37 (1.1–22 µM, 24 h) enhances *TNF-α*, *IL-10* and *IL-8* gene expression and secretion in immune cells, including monocytes [100,101]. Moreover, the LL-37-induced IL-8 production has been shown to depend on PI3K, MAPK (ERK1/2 and p38) signaling pathways and to involve transcription factors such as AP-1, AP-2, CREB, and E2F1 [54,101]. Whilst the KR-20 peptide almost abolished basal gene expression of *IL-10*, *TNF-α*, *IL-8*, and *IL-1*β (Figure S4). To our knowledge, this is the first report showing that KR-20 can regulate the relative expression of innate immune genes in monocytes.

LL-37 is known to modulate inflammatory responses across several immune cell types [47,98,99,100,102]. In our model, this peptide inhibited the *T. vaginalis*-induced upregulation of *IL-8*, *IL-1β*, and *COX-2* in monocytes (Figure 5), consistent with previous evidence showing its ability to dampen pathogen-driven inflammation. For instance, LL-37 (2.2 µM, 24 h) reduces the mRNA expression of *IL-18*, *CXCL10*, and *CCL2* in *Porphyromonas gingivalis* LPS-stimulated fibroblast [102], and pretreatment of macrophages with LL-37 (10 and 20 µM, 1 h) decreases the secretion of TNF-α, IL-6, and MCP-1 following LPS activation [98]. Similarly, LL-37 pretreatment lowers *TNF-α* expression in macrophages infected with *Mycobacterium avium* [103]. However, contrasting findings have also been reported; in monocyte-derived macrophages infected with *Mycobacterium tuberculosis*, LL-37 (0.2–3.3 µM, 4–24 h) increases the *IL-1β* gene expression [100]. These observations illustrate that LL-37 exerts context-dependent immunomodulatory effects shaped by its concentration, exposure time, cell type, and the nature of the infectious stimulus.

Several LL-37-derivative peptides and their analogs have shown to counteract inflammatory signaling in immune cells (e. g. IG-19, FK-13, and KR-12 analogs) [98,99]. In this sense, IG-19 and its analogs (5 µM) reduce LPS-induced IL-8 and TNF-α production in monocytes [104], while FK-13 analogs (5 and 10 µM) similarly suppress TNF-α secretion, NO production, and *iNOS* gene expression in macrophages [99]. Comparable anti-inflammatory effects have been reported for KR-12 analogs [98]. Those reports are in accordance with our results, KR-20 markedly attenuated the *T. vaginalis*-induced upregulation of *TNF-α*, *IL-8*, and *IL-1β* by *T. vaginalis* (Figure 6). This study was designed as an initial approach to evaluate whether KR-20 could modulate the immune response of monocytes during trichomoniasis. Although the results demonstrate a clear anti-inflammatory profile, the specific molecular mechanism underlying this effect remains to be elucidated. Future research will focus on determining whether KR-20 acts through signaling pathways previously associated with LL-37, such as MAPK, NF-κB, or PI3K-Akt, or through receptor-mediated modulation of innate immune responses.

Importantly, KR-20 achieves this modulatory effect within the context of a parasitic infection model—an area where LL-37 derivatives have been far less explored. This is, to our knowledge, the first report indicating that KR-20 reverses *T. vaginalis*-induced changes in the relative expression of innate immune genes in monocytes, highlighting its distinctive and promising immunomodulatory profile.

## 4. Materials and Methods

### 4.1. Cell Culture

The U937 monocyte cell line (ATCC CRL-1593.2) was kindly provided by Dr. Pablo César Ortíz Lazareno. The cell line was cultured at 37 °C for 24 h in a 5% CO_2_ atmosphere in culture bottles containing RPMI-1640 medium (Gibco Life Technologies, Grand Island, NY, USA, Cat. No. 31800-022) supplemented with 10% fetal bovine serum. Confluent cells were centrifuged at 375× *g* for 5 min, resuspended in phosphate-buffered saline, and their viability was determined by trypan blue exclusion. The pellet was then resuspended in RPMI-1640, and the number of cells required was adjusted according to the experiment.

### 4.2. Parasite Culture

The *T. vaginalis* GT-13 strain, was kindly donated by Dr. Felipe Padilla-Vaca and Dr. Fernando Anaya-Velázquez from the University of Guanajuato [41]. The use of this strain for evaluating the immunomodulatory and trichomonacidal effects of antimicrobial peptides was approved by the Institutional Bioethics Committee for Research at the University of Guanajuato under the protocol code CIBIUG-P32-2019. The parasite was grown at 37 °C for 24 h in screw-capped tubes containing the TYI-S-33 medium with 6% bovine serum at pH 7.0. The culture tubes were incubated at 4 °C for 10 min to harvest the trophozoites. The cells were collected and washed with phosphate-buffered saline by centrifugation at 375× *g* for 5 min. The trophozoites were then counted for the corresponding assay.

For the detection of *Mycoplasma* sp. in *T. vaginalis* (10 × 10^7^ trophozoites), genomic DNA was isolated using the Wizard^®^ Genomic DNA Purification Kit (Promega, Madison, WI, USA, Cat. No. TM050) according to the manufacturer’s instructions. DNA concentration and purity were determined using a NanoDrop 2000c spectrophotometer (Thermo Scientific, Wilmington, DE, USA). The presence or absence of *Mycoplasma* contamination was assessed with the Venor^®^ Mycoplasma Detection Kit (Sigma, St. Louis, MO, USA, Cat. No. MP0025), following the manufacturer’s protocol.

Regarding the detection of *Trichomonavirus*, total RNA was extracted using TRIzol™ Reagent (Invitrogen, Carlsbad, CA, USA, Cat. No. 15596026) according to the manufacturer’s instructions. RNA samples were treated with DNase I (Invitrogen, Cat. No. 18068-015), and cDNA synthesis was performed using the M-MLV Reverse Transcriptase Kit (Invitrogen, Cat. No. 28025013), following the manufacturer’s protocol. The resulting cDNA was quantified with a NanoDrop 2000c spectrophotometer (Thermo Scientific, Wilmington, DE, USA), and its integrity was verified by end-point PCR amplification of the constitutive gene *actin* (Table 1). A subsequent PCR was performed using *Trichomonavirus*-specific primers reported by El-Gayar et al. (2016) [105].

### 4.3. Antimicrobial Peptides

Synthetic peptides LL-37 (LLGDFERKSKEKIGKEFKRIVQRIKDFLRNLVPRTES) and KR-20 (KRIVQRIKDFLRNLVPRTES) were purchased from ISCA Biochemicals; the purity of all peptides was ≥95%, as analyzed by HPLC.

### 4.4. Antimicrobial Peptides Binding Assays to LPS

The binding capacity of LL-37 and KR-20 to bacterial lipopolysaccharide (LPS) was evaluated using an ELISA-based assay as previously described [106,107], with slight modifications. Medium-affinity plates (Immunolon 2HB, Rochester, NY, USA, Cat. No. 3455) were used, and 100 ng of LPS (Sigma, St. Louis, MO, USA, Cat. No. L3012) was added per well and incubated overnight at room temperature. Wells were then blocked with 10% (*w*/*v*) milk solution for 1 h. Different concentrations of AMPs (0, 1, 4, and 16 μg/mL) were added and incubated for 1 h at 4 °C. Then, wells were washed five times with PBS containing 0.05% Tween 20 (PBST), followed by incubation with rabbit anti-human LL-37 antibody (100 µL, 1:500; Santa Cruz, Dallas, TX, USA, Cat. No. sc-50423) for 1 h at room temperature. After washing with PBST, a peroxidase-linked goat anti-rabbit IgG secondary antibody (75 µL, 1:500; Abcam, Waltham, MA, USA, Cat. No. ab97080) was added and incubated for 1 h under the same conditions. Enzymatic activity was detected using 100 µL of freshly prepared ABTS substrate solution (0.03 M ABTS and 0.2% *v*/*v* 30% H_2_O_2_ in citrate-phosphate buffer). After 15 min of incubation at 37 °C, absorbance was measured at 450 nm using a microplate reader (Labsystems Multiskan MS, Vantaa, Finland).

### 4.5. Confocal Microscopy of the Interaction of Monocytes U937 and T. vaginalis

The U937 monocyte cell line (2 × 10^5^/well) and *T. vaginalis* (2 × 10^4^/well) were added to a 24-well culture plate containing coverslips in serum-free RPMI medium. The interaction was incubated for 3 h at 37 °C in a 5% CO_2_ atmosphere. Subsequently, the cells were fixed with 4% paraformaldehyde, and DNA was stained with 10 μg/mL Hoechst 33342 for 10 min. Coverslips were mounted with Prolong Gold (Invitrogen, Cat. No. P36930) and observed under a confocal microscope (Carl Zeiss LSM700, Jena, Germany).

### 4.6. Effect of the Antimicrobial Peptides KR-20 and LL-37 on U937 Cell Metabolic Activity

U937 cells (1 × 10^5^ cells/well) were cultured in 96-well polypropylene flat-bottom plates using serum-free RPMI without phenol red (Gibco Life Technologies, Grand Island, NY, USA, Cat. No. 118350530). Next, *T. vaginalis* (1 × 10^4^ trophozoites/well) and the antimicrobial peptides LL-37 or KR-20 (2.5, 5, and 10 μM) were added. The tripartite interaction was incubated 3 h in a humidified atmosphere containing 5% CO_2_. Monocyte metabolic activity was quantified as previously described [108,109]. Briefly, 10 μL of MTT [3-(4,5-dimethyl-2-thiazolyl)-2,5-diphenyl-2H-tetrazolium bromide, Sigma Cat. M2128] at 5 mg/mL in PBS was added to each well and incubated at 37 °C for 4 h. Subsequently, 150 μL of 2-propanol/1 M HCl (19:1 *v*/*v*) was added per well to dissolve the formazan crystals. Absorbance was measured at 570 nm using a microplate spectrophotometer (Labsystems Multiskan MS).

### 4.7. Detection of Nitric Oxide Production by U937 Cells

For the interaction assays, U937 monocytes with *T. vaginalis* were co-cultured at a multiplicity of infection (MOI) of 10:1 (monocytes:trophozoite) in polypropylene flat-bottom microplates containing serum-free RPMI medium without phenol red (Gibco Life Technologies, 118350530), together with LL-37 (2.5 or 5 µM) or KR-20 (2.5, 5 or 10 µM). After 3 h of incubation, the conditioned medium (CM) was collected to indirectly determine nitric oxide (NO) secreted by monocytes using the Griess reaction, as previously described [110].

The Griess reagent was prepared by mixing equal volumes of solution A (10% sulfanilamide, 40% H_3_PO_4_, Sigma) and solution B (1% N-(1-naphthyl) ethylenediamine dihydrochloride, Sigma). Then, 20 µL of Griess reagent was mixed with 140 µL of CM and the absorbance was measured at 550 nm. Nitrite (NO_2_^−^) concentration was calculated using a sodium nitrite (NaNO_2_, pH 7.4, Sigma) standard curve ranging from 1 to 100 µM. Monocytes and *T. vaginalis* cultured separately were included as negative controls.

### 4.8. RNA Isolation Monocytes and Analysis of the Innate Immune Relative Gene Expression

For RNA isolation, 2 × 10^5^ U937 monocytes, 2 × 10^4^ *T. vaginalis* trophozoites, and the peptides LL-37 (2.5 or 5 µM) or KR-20 (2.5–10 µM) were co-incubated in 24-well polypropylene flat-bottom plates containing serum-free RPMI medium without phenol red (Gibco, Cat. No. 118350530). After a 3 h incubation period, total RNA isolation was performed using Trizol™ Reagent (Invitrogen, Cat. No. 15596026) according to the manufacturer’s instructions and subsequently used for cDNA synthesis. Genomic DNA contamination was removed from RNA samples with DNase I treatment (Invitrogen, Cat. No. 18068-015). A reverse transcription reaction was performed in 20 μL containing 25 μg/mL Oligo d(T) (Invitrogen, Cat. No. 18418012) and 500 nM dNTPs (Invitrogen, Cat. No. 10297-018). The reaction was incubated at 65 °C for 5 min and immediately transferred to ice. Then, 1X first-strand buffer (Invitrogen, Cat. No. 28025013), 10 mM dithiothreitol, and 2 U/μL RNase inhibitor (Invitrogen, Cat. NO. 10777-019) were added to the reaction mixture and incubated at 37 °C for 2 min. Finally, 10 U/μL M-MLV reverse transcriptase (Invitrogen, Cat. No. 28025013) was added, and the mixture was incubated at 37 °C for 50 min, followed by 70 °C for 15 min.

Relative quantification of gene expression was performed using the comparative Ct method (ΔΔCt) on a StepOnePlus Real-Time PCR System (Applied Biosystems, Foster City, CA, USA) according to the manufacturer’s instructions. The reactions were performed using a SYBR Green PCR Master Mix (PCRBiosystems, London, ENG, United Kingdom, PB20.16). Specific primers were used to amplify genes encoding interleukins and chemokines (Table 1). GAPDH was used as internal controls (endogenous genes).

**Table 1:** Sequences of the primers used in this study.

Specificity	Primer	Sequence (5′-3′)	Fragment Size (bp)	Tm (°C)	References
IL-8	F	CAGTTTTGCCAAGGAGTGCTAA	225	62	This study
	R	TCTCAGCCCTCTTCAAAAACTTCTC			
IL-1β	F	ATGATGGCTTATTACAGTGGCAA	132	62	This study
	R	GTCGGAGATTCGTAGCTGGA			
TNF-α	F	CCCTGGTATGAGCCCATCTATC	120	66	[111]
	R	AAAGTAGACCTGCCCAGACTCG			
IL-10	F	GGTGACACACTATGGTATTTGAGTG	174	64	This study
	R	CAAGCCCAGAGACAAGATAAATTAG			
TGF-β	F	CGGCAGCTGTACATTGACTTC	129	64	This study
	R	CTTGCTGTACTGCGTGTCCA			
COX-2	F	AATGGGGTGATGAGCAGTTGTTC	202	62	This study
	R	GGATGCCACTGATAGAGGGTGTTA			
GAPDH	F	GACAGTCAGCCGCATCTTCT	127	64	[112]
	R	TTAAAAGCAGCCCTGGTGAC			
TvActin	F	TTAAAAGCAGCCCTGGTGAC	451	55	[113]
	R	TGTCGGCCGTCCAAAGTA			

### 4.9. Statistical Analysis

Statistical analyses were performed using the Shapiro–Wilk normality test (http://sdittami.altervista.org/shapirotest/ShapiroTest.html, accessed on 30 July 2022). We used the GraphPad Prism 8.0.1 package to perform a one-way analysis of variance for data with a normal distribution and a Kruskal–Wallis test for non-parametric data.

## 5. Conclusions

Collectively, our findings demonstrate that KR-20 preserves monocyte metabolic activity, reduces parasite-nitric oxide production more effectively than LL-37, and attenuates the transcription of key proinflammatory genes while retaining the LPS-binding capacity characteristic of the parent peptide. These results identify KR-20 as a promising immunomodulatory peptide with potential applicability in the context of trichomoniasis. Although this works represents an initial approximation and focuses on transcriptional responses, future studies assessing protein-level inflammatory mediators and the molecular pathways underlying KR-20 activity will be essential to fully elucidate its therapeutic potential.

## Data Availability

Data set available on request from the authors.

## Supplementary Materials

The following supporting information can be downloaded at https://www.mdpi.com/article/10.3390/molecules31030413/s1. Figure S1: Metabolic effect of antimicrobial peptides LL-37 and KR-20 on monocyte activity; Figure S2: Nitric oxide production by monocytes incubated with antimicrobial peptides; Figure S3: Effect of LL-37 on innate immune gene expression in U937 monocytes; Figure S4: Effect of KR-20 on innate immune gene expression in monocytes; Figure S5: Detection of *Mycoplasma* sp. in *T. vaginalis*; Figure S6: Evaluation of Trichomonavirus presence in *T. vaginalis*.

## Abbreviations

The following abbreviations are used in this manuscript:

AMPs	Antimicrobial peptides
HIV	Human Immune Virus
KR-20	Antimicrobial peptide derivative LL-37
LL-37	Human cathelicidin
LPS	Lipopolysaccharide
MOI	Multiplicity of infection
MTZ	Metronidazole
NO	Nitric Oxide
STI	Sexually Transmitted Infection
TVV	*Trichomonas vaginalis* virus
