# Supplementary material for: Immunomodulatory Effects of the Antimicrobial Peptide KR-20: Implications for Trichomoniasis
Source: Molecules. 2026 Jan 26;31(3):413. doi: 10.3390/molecules31030413 (PMC12898638; doi:10.3390/molecules31030413)
Supplement: Supplementary file 1 [file molecules-31-00413-s001.zip › molecules-3931118-supplementary.pdf]

Supplementary Materials:

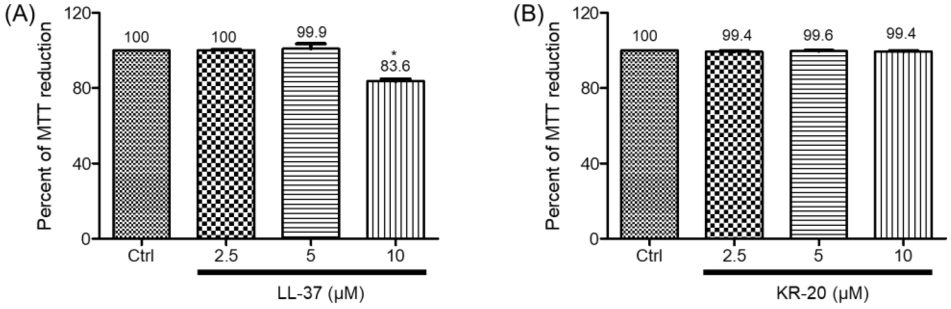

Figure S1. Metabolic effect of antimicrobial peptides LL-37 and KR-20 on monocyte metabolic activity. U937 monocytes were cultured with (A) LL-37 or (B) KR-20 for 3 h, and metabolic activity was determined by MTT assay. Bars indicate the mean ± SE obtained from four independent experiments; each performed in triplicate. Statistical analysis was conducted using the Kruskal-Wallis test, comparing each experimental condition with unstimulated control cells (* $p<0.05$).

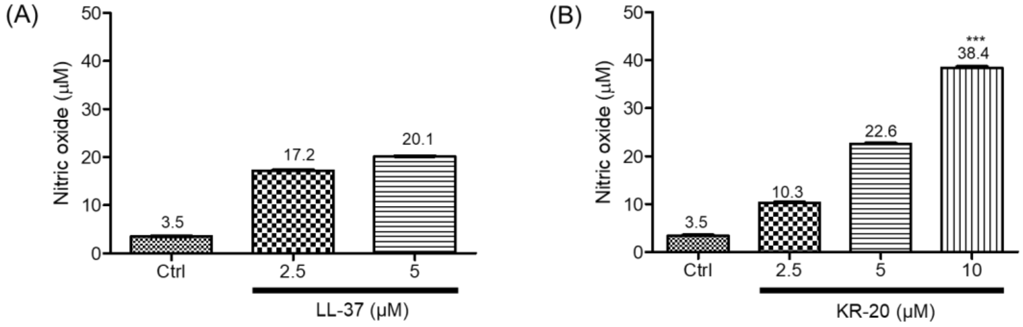

Figure S2. Nitric oxide production by monocytes incubated with antimicrobial peptides. U937 cells were incubated for 3 h at 37°C with the antimicrobial peptide (A) LL-37 or (B) KR-20 at different concentrations. Supernatants were collected, and nitrites were quantified using the Griess reaction. Each bar represents the mean ± SE of four independent experiments performed in triplicate. Kruskal-Wallis statistical analysis was carried out, comparing each experiment condition with unstimulated control cells (*** $p<0.001$).

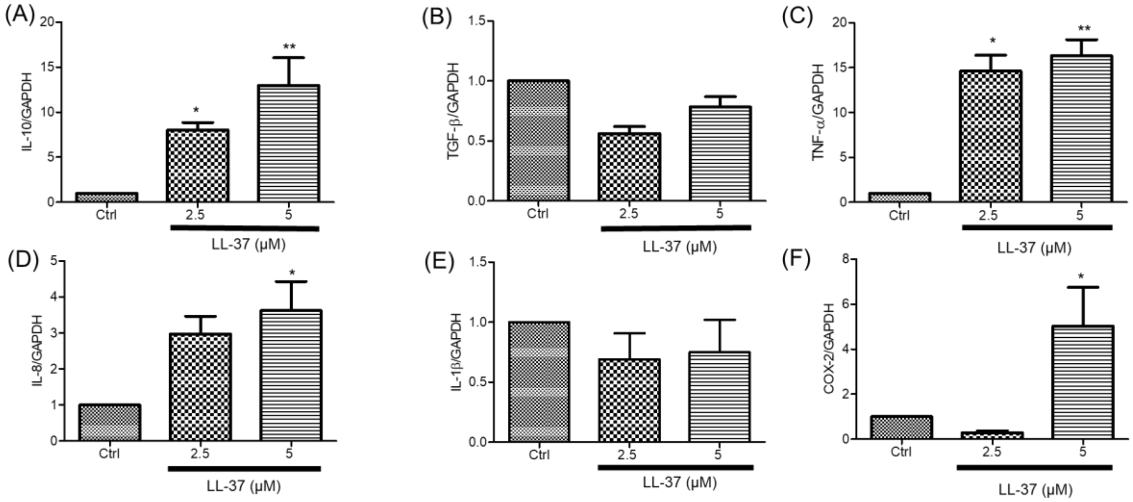

Figure S3. Effect of LL-37 on innate immune gene expression in U937 monocytes. U937 cells were incubated for 3 h at 37°C with LL-37 (2.5 or 5 μM). LPS (1 μg/mL) was used as a positive control. Gene expression levels were quantified using the ΔΔCt method and normalized to *GAPDH*. Panels represent: (A) *IL-10*, (B) *TGF*-β, (C) *TNF-α*, (D) *IL-8*, (E) *IL-1*β, and (F) *COX-2*. Bars correspond to the mean ±SE of three experiments, each performed in triplicate. The results were analyzed using the Kruskall-Wallis test. The symbol "*" indicates significant differences in relation to unstimulated control cells (* $p<0.05$, ** $p<0.01$).

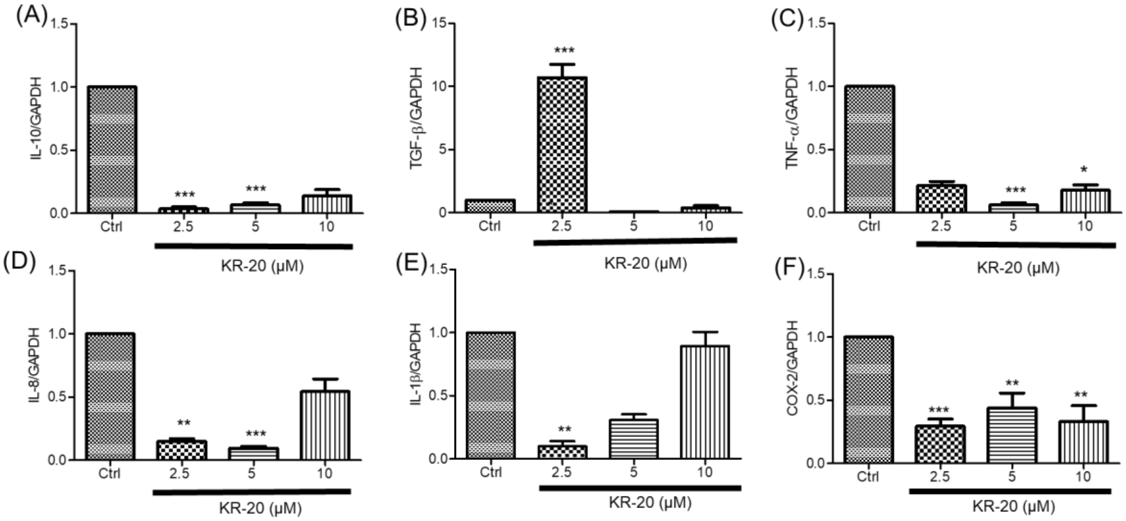

Figure S4. Effect of KR-20 on innate immune gene expression in monocytes. U937 cells were incubated for 3 h at 37°C with KR-20 (2.5, 5, or 10 μM). LPS (1 μg/mL) was included as a positive control. Gene expression levels were quantified using the ΔΔCt method and normalized to *GAPDH*. Panels correspond to (A) *IL-10*, (B) *TGF*-β, (C) *TNF-α*, (D) *IL-8*, (E) *IL-1*β, and (F) *COX-2*. Values represent the mean ±SE from three independent experiments conducted in triplicate. Statistical analysis was performed using Kruskall-Wallis test. The symbol "*" denotes significant differences in relation to unstimulated cells (* $p<0.05$, ** $p<0.01$, *** $p<0.001$).

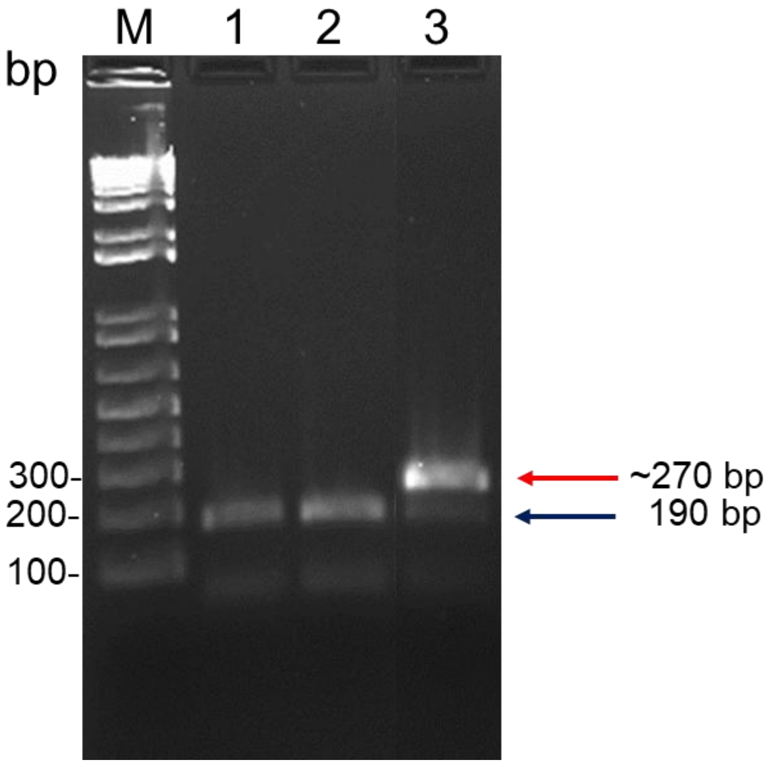

Figure S5. Detection of *Mycoplasma* sp. in *T. vaginalis***.** The Venor *Mycoplasma* kit was used for the detection of this bacterium. (M) 1 Kb marker plus DNA ladder; (1) Internal PCR control (190 bp); (2) GT-13 DNA; (3) Positive control (~270 bp). 1% agarose gel, stained with ethidium bromide.

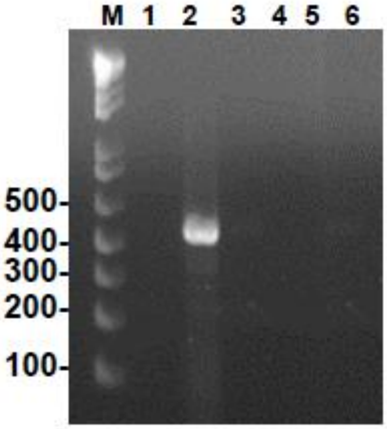

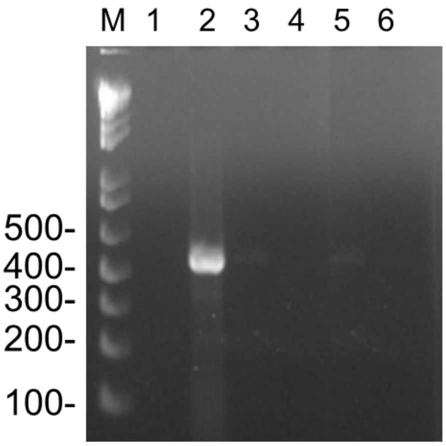

Figure S6. Evaluation of Trichomonavirus presence in *T. vaginalis*. An end-point PCR was performed using cDNA synthesized from GT-13 strain to identify Trichomonavirus. (M) 1 Kb plus DNA ladder; (1) PCR negative control; (2) *T. vaginalis* actin (451 bp). Expected amplicon sizes were: (3) Tvv1 ~569 bp, (4) Tvv2 ~625 bp, (5) Tvv3 ~437 bp, and (6) Tvv4 ~514 bp. PCR products were resolved on 1% agarose gels stained with ethidium bromide.
